# Diagnosis of Queensland Tick Typhus and African Tick Bite Fever by PCR of Lesion Swabs

**DOI:** 10.3201/eid1506.080855

**Published:** 2009-06

**Authors:** Jin-Mei Wang, Bernard J. Hudson, Matthew R. Watts, Tom Karagiannis, Noel J. Fisher, Catherine Anderson, Paul Roffey

**Affiliations:** Royal North Shore Hospital, St. Leonards, Sydney, Queensland, Australia (J.-M. Wang, B.J. Hudson, M.R. Watts, T. Karagiannis, N.J. Fisher); Woodland Street Medical Practice, Balgowlah, New South Wales, Australia (C. Anderson); Charles Sturt University, Wagga Wagga, New South Wales, Australia (P. Roffey)

**Keywords:** Rickettsia, spotted fever, PCR, swabs, Queensland, Australia, tick, vector-borne infections, dispatch

## Abstract

We report 3 cases of Queensland tick typhus (QTT) and 1 case of African tick bite fever in which the causative rickettsiae were detected by PCR of eschar and skin lesions in all cases. An oral mucosal lesion in 1 QTT case was also positive.

Queensland tick typhus (QTT) is endemic to Sydney, New South Wales, Australia (*1*–*6*). A prospective study of 80 serologically confirmed QTT cases (63 acquired in Sydney) yielded 68 cases with rash, of which 62% and 27%, respectively, were vesicular and pustular (*6*). This study yielded an isolate of *Rickettsia* *australis* from a patient who acquired QTT in Sydney (*1*). Eschar biopsies and removed eschars have been used for PCR confirmation of rickettsial and scrub typhus infections (*7*). We report 3 cases of QTT and 1 case of African tick bite fever (ATBF) in which the causative rickettsiae were detected by PCR of eschar and skin lesions in all cases and by an oral mucosal lesion in one of the QTT cases. Clinical details of the cases are summarized below.

## The Cases

### Case 1

A 45-year-old woman sought treatment at the emergency department in one of our district hospitals after being bitten by a tick in suburban Sydney. Clinical signs were inguinal eschar, tender local lymphadenopathy, fever to 39.8°C, severe headache, myalgia, arthralgia, and generalized sparse rash characterized by maculopapular and vesiculopustular lesions.

### Case 2

A 32-year-old woman, a resident of suburban Sydney, sought treatment for an acute febrile illness (fever to 41°C), severe headache, myalgia, arthralgia, and rash. She did not recall a tick bite. Clinical examination showed an eschar on the torso, generalized sparse rash characterized by maculopapular and vesiculopustular lesions, plus oral mucosal lesions and a palatal lesion.

### Case 3

A 79-year-old woman sought treatment for gradual onset of fever (to 38.8°C) after being bitten by a tick at her home in suburban Sydney. Examination showed an eschar on the scalp, tender local lymphadenopathy, and generalized rash with maculopapular and vesicular components.

In these 3 cases, acute-phase serologic results were negative; convalescent-phase serologic results yielded an *R. australis* indirect fluorescent antibody (IFA) titer of 512. Illness resolved completely after treatment with doxycycline, 100 mg 2×/day, for 14 days.

### Case 4

A 57-year-old man with gradual onset of fever (to 38.8°C) sought treatment 10 days after visiting a game park in South Africa. He did not recall a tick bite. Clinical examination showed an eschar on the torso, tender local lymphadenopathy, and generalized sparse rash characterized by papular and vesiculopustular lesions. Illness resolved completely after a regimen of doxycycline, 100 mg 2×/day. *R. australis* IFA acute titer was <128; titer for serum collected 44 days after fever onset was 128. *R. africae* antigen was unavailable for IFA. Acute-phase and convalescent-phase serum samples were negative for *R. typhi* and *Orientia tsutsugamushi* (all IFA titers <128).

Specimens on all 4 cases were collected as shown in the Table. Serologic analysis was performed by using the reference method of IFA testing using *R. australis* infected cells, according to Philip et al (*8*). Total antibody to *R. australis* was detected. A titer of 128 was regarded as borderline; titers >128 were regarded as positive. To confirm recent infection, a 4-fold rise in titer is preferre .

**Table:** PCR results by specimen, relation to clinical signs, and doses of doxycycline taken, Australia*

Case no.	Specimen type	Duration of lesion, d	Duration of fever, d	No. 100-mg doxycycline doses	PCR result
1					
EDTA	Blood, EDTA	NA	3	2	
Eschar	Eschar, Amies, dry	6	3	2	
Eschar	Eschar, virus, dry	6	3	2	+
Vesicle	Vesicle, virus, dry	6	3	2	+
Vesicle	Vesicle, virus, dry	7	4	4	–
2					
Blood	Blood, EDTA	NA	3	0	–
Eschar	Eschar, Amies, dry	6	3	0	+
Eschar	Eschar, virus, dry	8	5	0	+
Eschar	Eschar, virus, saline	10	7	4	+
Vesicle	Vesicle, Amies, dry	1	3	0	–
Vesicle	Vesicle, virus, dry	3	5	0	–
Vesicle	Vesicle, virus, saline	5	7	4	–
Palatal lesion	Lesion, virus, dry	5	7	4	+
3					
Blood	Blood, EDTA	NA	1	0	–
Eschar	Eschar, virus, saline	10	2	1	+
Vesicle	Vesicle, virus, saline	6	2	1	+
4					
Blood	Blood, EDTA	NA	5	2	–
Eschar	Eschar, virus, dry	10	5	2	+
Vesicle	Vesicle, virus, dry	4	5	2	+
Vesicle	Vesicle, virus, dry	4	5	2	+

*NA, not applicable.

For PCR, specimens were collected by swab of eschar margin or, in the case of unroofed vesicular lesions, in the manner described by Rawls (*9*). Copan Amies Transport swab without charcoal and Copan Virus Transport swab (Interpath Services, Melbourne, Victoria, Australia) were used. Duplicates were collected from the same lesion in some cases; 1 specimen was collected using a dry swab, and another by using a sterile saline moistened swab.

Identification of *Rickettsia* spp. citrate synthase-encoding gene (*gltA*) PCR was performed based on the method of Roux et al. (*10*). DNA was extracted from the specimens by using the QIAamp DNA Mini kit (QIAGEN, Doncaster, Victoria, Australia) according to manufacturer’s protocol. Positive control was *R. australis* DNA extracted from 3–5-day-old Vero cell culture, serially diluted from 1:10 to 1:105. Extraction blanks, consisting of water processed along with the specimens, were also included as negative controls.

Eppendorf DNA Thermalcycler (Eppendorf, North Ryde, New South Wales, Australia) was used for all PCR amplification. Five microliters of each DNA extraction was added to 45 µL of master mixture for each reaction. Final reagent concentration was 10 pmol for each primer, 200 µM for each deoxynucleotide triphosphate, 1 U of AmpliTaq Gold DNA Polymerase (Applied Biosystems, Roche Molecular System, Inc., Indianapolis, IN, USA), and 1× PCR buffer. The following thermal cycler parameters were used with the primer pairs for the *gltA* gene RpCS.877p and Rp1273r primers, which amplify a 397-bp portion of the *gltA* gene from all *Rickettsia* spp.: 95°C (10 min), followed by 40 cycles of 95°C (30 s), 45°C (30 s), and 72°C (55 s), followed by an extension period (72°C, 10 min).

The amplicons were detected after electrophoresis on a 4% agarose gel stained with ethidium bromide and purified using the QIAquick DNA Purification kit (QIAGEN) for sequencing analysis. BLAST (www.ncbi.nlm.nih.gov/BLAST) was used for comparison and analysis of sequence data obtained. PCR-positive specimens on QTT cases yielded sequences 99% homologous with *R. australis* (Phillips strain). Amplification was unsuccessful in all negative controls. PCR results for herpes simplex and varicella zoster viruses were both negative on the swab of the palatal lesion. PCR-positive specimens on the ATBF case yielded sequences that were 99% homologous with *R. africae*. GenBank accession nos. for sequences are EU543436 (case 1 eschar); EU543438 (case 2 palatal lesion); EU543438 (case 3 vesicular lesion); and EU714268 (case 4 eschar).

PCR results for blood specimens were negative in all cases. For QTT cases, all eschars yielded positive PCR results for *R. australis*, irrespective of type of swab used. Vesicle swabs also yielded positive PCR results for *R. australis* but only in cases 1 and 3. In case 2, the palatal lesion yielded a positive PCR result for *R. australis*. For the ATBF case, all vesicular lesions and eschar swabs yielded a positive PCR result for *R. africae*.

Based on previous reports and recommendations, tissue collected from the eschar site is the most likely specimen to provide a positive result for rickettsia PCR or culture (*11*,*12*). Lepidi et al. (*13*) reported 8 cases of ATBF wherein age of eschars at biopsy ranged from 5 to 10 days. Of 8 eschar biopsies, positive results were obtained by immunohistochemical analysis (IHC) (6/8), serologic analysis (4/8), culture (4/8), regular PCR (6/8), and nested PCR (8/8). Although we did not use nested PCR, we still had 100% detection of rickettsiae in 4 eschars of similar ages and after up to 4 doses of doxycycline, 100 mg. Eschar scab in scrub typhus yielded positive nested PCR result for *O.* *tsutsugamushi* in a child 7 days after commencement of treatment with azithromycin (*7*).

Accordingly, eschar specimens may permit characterization of the causative organism even after days of antimicrobial drug therapy. In the cases we studied, no blood specimens were positive by the PCR method used; swabs of all eschars and some vesicular skin lesions were positive. Rickettsemia may not be detected by any method because of variables such as stage of illness, effective antimicrobial drug therapy, individual variation in level of rickettsemia, and sensitivity of the detection method. We acknowledge that insufficient numbers of rickettsiae for detection by the PCR method used may have resulted in some false negative results. Real-time PCR methods likely offer greater sensitivity while also providing quantitative data. Additionally, PCR-negative lesions may have been positive by IHC, which when used in conjunction with PCR, has improved rickettsiae detection in skin biopsies (*12*).

Eschar or vesicular skin lesion swab has high patient acceptance because the test is simple and virtually painless. Although lesion biopsy enables IHC (and improved rickettsiae detection), our results justify further exploration of swab PCR to confirm diagnosis of QTT, ATBF, and other rickettsial spotted fevers.
